# Air Pollution and Inflammation (Interleukin-6, C-Reactive Protein, Fibrinogen) in Myocardial Infarction Survivors

**DOI:** 10.1289/ehp.10021

**Published:** 2007-04-18

**Authors:** Regina Rückerl, Sonja Greven, Petter Ljungman, Pasi Aalto, Charalambos Antoniades, Tom Bellander, Niklas Berglind, Christina Chrysohoou, Francesco Forastiere, Bénédicte Jacquemin, Stephanie von Klot, Wolfgang Koenig, Helmut Küchenhoff, Timo Lanki, Juha Pekkanen, Carlo A. Perucci, Alexandra Schneider, Jordi Sunyer, Annette Peters

**Affiliations:** 1 GSF National Research Centre for Environment and Health, Institute of Epidemiology, Neuherberg, Germany; 2 Ludwig-Maximilians University, Department of Statistics, Munich, Germany; 3 Institute of Environmental Medicine, Karolinska Institute, Stockholm, Sweden; 4 Department of Physical Sciences, University of Helsinki, Helsinki, Finland; 5 Department of Hygiene and Epidemiology, University of Athens, Athens, Greece; 6 Department of Occupational and Environmental Health, Stockholm County Council, Stockholm, Sweden; 7 Local Health Authority, Department of Epidemiology, Rome, Italy; 8 Municipal Institute of Medical Research (IMIM), Barcelona, Spain; 9 Department of Internal Medicine II, Cardiology, University of Ulm Medical Center, Ulm, Germany; 10 Environmental Epidemiology Unit, National Public Health Institute (KTL), Kuopio, Finland; 11 School of Public Health and Clinical Nutrition, University of Kuopio, Kuopio, Finland; 12 Focus-Network Aerosols and Health, GSF National Research Centre for Environment and Health, Neuherberg, Germany

**Keywords:** air pollution, C-reactive protein, CRP, epidemiology, fibrinogen, IL-6, inflammation, myocardial infarction, ultrafine particles

## Abstract

**Background:**

Numerous studies have found that ambient air pollution has been associated with cardiovascular disease exacerbation.

**Objectives:**

Given previous findings, we hypothesized that particulate air pollution might induce systemic inflammation in myocardial infarction (MI) survivors, contributing to an increased vulnerability to elevated concentrations of ambient particles.

**Methods:**

A prospective longitudinal study of 1,003 MI survivors was performed in six European cities between May 2003 and July 2004. We compared repeated measurements of interleukin 6 (IL-6), fibrinogen, and C-reactive protein (CRP) with concurrent levels of air pollution. We collected hourly data on particle number concentrations (PNC), mass concentrations of particulate matter (PM) < 10 μm (PM_10_) and < 2.5 μm (PM_2.5_), gaseous pollutants, and meteorologic data at central monitoring sites in each city. City-specific confounder models were built for each blood marker separately, adjusting for meteorology and time-varying and time-invariant covariates. Data were analyzed with mixed-effects models.

**Results:**

Pooled results show an increase in IL-6 when concentrations of PNC were elevated 12–17 hr before blood withdrawal [percent change of geometric mean, 2.7; 95% confidence interval (CI), 1.0–4.6]. Five day cumulative exposure to PM_10_ was associated with increased fibrinogen concentrations (percent change of arithmetic mean, 0.6; 95% CI, 0.1–1.1). Results remained stable for smokers, diabetics, and patients with heart failure. No consistent associations were found for CRP.

**Conclusions:**

Results indicate an immediate response to PNC on the IL-6 level, possibly leading to the production of acute-phase proteins, as seen in increased fibrinogen levels. This might provide a link between air pollution and adverse cardiac events.

Ambient air pollution has been associated with cardiovascular mortality (Forastiere et al. 2005; Peters et al. 2000; Schwartz and Dockery 1992) and hospital admissions for various cardiovascular diseases (Burnett et al. 1997; Schwartz 1999). Also, an elevated risk for acute myocardial infarction (MI) (Lanki et al. 2006; Peters et al. 2001a) and cardio-respiratory symptoms (de Hartog et al. 2003) has been reported in relation to air pollution. Some studies have suggested that patients with preexisting coronary heart disease (CHD) (Goldberger et al. 2001) might be a particularly susceptible population.

The exact mechanisms linking the inhalation of ambient air particles to an acute exacerbation of cardiovascular disease are not completely understood (Brook et al. 2004). Alveolar inflammation induced by particles may either directly or via oxidative stress lead to systemic inflammation with increased levels of blood coagulability, progression of atherosclerosis, and destabilization or even rupture of vulnerable plaques, resulting in acute ischemic events (Brook et al. 2004; Seaton et al. 1995).

So far, studies using repeated measures to assess the association between ambient air particles and inflammatory markers have had controversial results. In addition, they have been conducted only on a small scale, with samples sizes ranging from 9 to 112 (Riediker et al. 2004; Ruckerl et al. 2006; Seaton et al. 1999). In larger studies, however, associations have been based on single blood measurements (Zeka et al. 2006), and the examined populations have encompassed healthy and diseased subjects, covering a variety of diseases. All these differences might explain the conflicting results.

For interleukin 6 (IL-6), hypothesized to play a central role in the triggering of the inflammatory process (Woods et al. 2000), associations with high levels of particulate matter (PM) < 10 μm in aerodynamic diameter (PM_10_) have been shown (van Eeden et al. 2001), although a study in elderly subjects in the United Kingdom (Seaton et al. 1999) did not reveal significant associations with ambient PM_10_. The present study was designed to address the responses of IL-6, fibrinogen, and C-reactive protein (CRP) to elevated air pollution levels in a large cohort of MI survivors across Europe. We were particularly interested in MI survivors because they are especially prone to a progression of atherosclerosis and adverse cardiovascular events.

## Materials and Methods

### Study population

A prospective longitudinal study of post-MI patients was performed in six European cities—Athens (Greece), Augsburg [Germany, KORA (Cooperative Health Research in the Augsburg Region) (Lowel et al. 2005)], Barcelona (Spain), Helsinki (Finland), Rome (Italy), and Stockholm (Sweden)—chosen to include a variety of geographic conditions and air pollution characteristics [see Appendix 1 for participants; see Supplemental Material (http://www.ehponline.org/docs/2007/10021/suppl.pdf) for data]. The study design is described in detail elsewhere (Peters et al., in press). In brief, we recruited patients 35–80 years of age who had experienced an MI between 4 months and 6 years before the start of the study. Patients with MI or interventional procedures < 3 months before the beginning of the study or with chronic recurring inflammatory diseases such as Crohn’s disease were not included.

Preferably, current nonsmokers were recruited. All partners approved the study protocol at their local human subjects committees, and written informed consent was obtained from all patients. All methods used in the study centers were conducted according to common standard operating procedures.

### Clinical measurements

Patients were invited to participate in six to eight clinical visits between May 2003 and July 2004. The visits were scheduled every 4–6 weeks on the same weekday and at the same time of the day to minimize the impact of weekly and circadian variation. At the first visit, a baseline questionnaire was administered regarding health status, medication intake, and smoking history. Blood pressure and body mass index (BMI) were measured and a blood serum sample was drawn to assess baseline serum lipids, glycosylized hemoglobin (HbA1c; an indicator of diabetic status) and N-terminal proB-type natriuretic peptide (NT-proBNP; an indicator for left ventricular dysfunction).

At each clinical visit a 7-day recall on medication intake was obtained. Venous ethylene-diamine tetraacetic acid (EDTA)–plasma samples were collected for the determination of the inflammatory markers. Samples were cooled and stored at 4°C until further processing within a maximum of 4 hr. The EDTA-blood was centrifuged at 4°C in a precooled centrifuge for 20 min at 2,500 × *g*. Plasma aliquots were shipped on dry ice to the central laboratory in Ulm, Germany, and were stored at –80°C until analysis. Blood samples were analyzed by means of a commercial enzyme-linked immunosorbent assay (ELISA) for IL-6 (quantitative high sensitive IL-6 immunoassay; RD Systems GmbH, Wiesbaden, Germany) and immunonephelometry for fibrinogen and high-sensitivity CRP (Dade Behring Marburg GmbH, Marburg, Germany). Because CRP and fibrinogen concentrations were measured by a fully automated assay, only single measurements were available, except for results above and below the detection limit, which were double-checked. Within- and between-patient variability for a number of blood samples that were tested as quality assurance measures are described elsewhere (Peters et al., in press).

### Air pollution and meteorologic data

Air pollution data from fixed monitoring sites representing urban background concentrations were collected for each city according to standard procedures already employed in several European studies of air pollution (Aalto et al. 2005; Katsouyanni et al. 1996). We obtained hourly means of particles [black smoke (BS), black carbon (BC), mass concentration of PM_10_, and mass concentration of particles < 2.5 μm in diameter (PM_2.5_)], gaseous air pollutants (carbon monoxide, sulfur dioxide, ozone, nitric oxide, nitrogen dioxide) and meteorologic variables (air temperature, relative humidity, barometric pressure, dew point temperature) through city-specific air monitoring networks and meteorologic services. If data were recorded locally at smaller units, at least 50% of the data for 1 hr needed to be present for the hourly value to be considered useable. For valid 8- or 24-hr mean values, at least 75% of the observations needed to be present. Particle number concentration (PNC) measurements as indicator for ultrafine particles were performed using condensation particle counters (CPC; 3022A; TSI, Shoreview, MN, USA) in all centers.

Missing data on the aggregate level were replaced using a formula adapted from the APHEA (Air Pollution and Health—A European Approach) method (Katsouyanni et al. 1996) [see Supplemental Material (http://www.ehponline.org/docs/2007/10021/suppl.pdf)]. We calculated apparent temperature by using the formula of Steadman (1984) and Kalkstein and Valimont (1986).

We used moving averages of ambient concentrations of air pollutants and meteorogic variables to characterize the exposures by calculating the individual 24-hr average exposure for each person immediately preceding the clinical visit (lag 0) up to 4 days (lag 1–lag 4). In addition, we calculated the mean of lags 0–4 for the air pollution data and the mean of lags 0 and 1, the mean of lags 2 and 3, the mean of lags 0–3, and the mean of lags 0–4 for the meteorologic variables, if at least half of the relevant lags were available.

### Statistical analyses

#### Analytical strategy

Given previous findings, we hypothesized that particulate air pollution induces systemic inflammation. Specifically, we assumed that IL-6 would increase in association with increased levels of ambient particle concentrations of the preceding or same day, because immediate effects on IL-6 have been shown before (van Eeden et al. 2001), and the cytokine has a very short half life (2–6 hr) (Riches et al. 1992).

We also hypothesized that an acute-phase response involving *de novo* synthesis of proteins in the liver would require an induction time of 1–2 days. This would translate to an increase in fibrinogen concentrations with elevated particle concentrations of the previous 5 days [half-life 2–3 days (Thomas 1998)] and an increase in CRP in association with ambient particle concentrations 2–3 days before blood withdrawal [half-life 19 hr (Koenig et al. 2003)]. Similar results have been shown in previous studies (Ruckerl et al. 2006; Seaton et al. 1999).

#### Statistical model

We analyzed data using mixed-effects models with random patient effects accounting for repeated measures. Because the half-lives of the markers were much shorter than the intervals between visits, we assumed a compound symmetry structure for the covariance matrix to model the correlation between repeated measures in each patient. Penalized splines (P-splines) in the additive mixed-model framework were used to allow for nonparametric exposure–response functions (Greven et al. 2006). IL-6 and CRP needed to be log-transformed to fulfill the model assumption of residual normality.

City-specific confounder models without air pollutants were built for each blood marker separately. In addition to potential time-varying confounders, we included time-invariant patient characteristics associated with the mean levels of inflammatory markers to permit the assumption of a normally distributed random patient intercept.

In a first step, time-invariant factors were selected for all cities combined. In the second step, for each city a more parsimonious model was selected out of the formerly chosen variables [see Supplemental Material, Table 1 (http://www.ehponline.org/docs/2007/10021/suppl.pdf)]. With this strategy, we adjusted for variables that influenced the mean levels of the respective blood markers in the single cities, such as age, sex, and BMI. These variables varied among the cities, possibly reflecting underlying differences in the populations across Europe as well as chance influences. To ensure sufficient adjustment for season and meteorology, long-term time trend and apparent temperature were forced into all models. Additionally, relative humidity, time of day, and day of the week were included if this adjustment improved the model fit. We considered lag 0, the mean of lags 0 and 1, the mean of lags 2 and 3, and the mean of lag 0–3 for the weather variables; for fibrinogen, we additionally assessed the mean of lags 0–4. P-splines were used to model continuous covariables and were compared with linear terms and polynomials of degrees 2 and 3. All decisions on goodness-of-fit were based on Akaike’s Information Criterion (AIC) (Akaike 1973). Only after this adjustment did we examine mean changes of the inflammatory markers in association with air pollution. Single air pollutants were added and effects estimated linearly. After the city-specific data analyses, we assessed heterogeneity between centers (Normand 1999). We combined city-specific effect estimates using meta-analysis methodology (Van Houwelingen et al. 2002). Additionally, we checked whether active smoking, levels of NT-proBNP > 80 pg/mL (de Lemos et al. 2003), and HbA1c > 6.5%, respectively, modified the effects of air pollution on blood parameters.

Data were analyzed using the statistical package SAS version 9.1 (SAS Institute Inc., Cary, NC, USA). Effect estimates are presented as percent change of geometric mean of the blood marker level (IL-6, CRP) and change of the arithmetic mean level (fibrinogen, percent of overall mean) together with 95% confidence intervals (CIs) based on an increase in air pollution concentrations from the first to the third quartile [interquartile range (IQR)].

### Sensitivity analyses

We performed sensitivity analyses to explore the robustness of the models by using a more parsimonious and an extended model. Also, indicator variables for season and for potential inflammation due to diseases or surgery shortly before the blood withdrawal were added to the model.

## Results

### Study population

Baseline characteristics of the study population are given in Table 1. In total, 1,003 MI survivors who had at least two valid repeated blood samples were taken into the analyses. These were 84% of the targeted 1,200 patients.

### Blood parameters

Of 6,068 collected blood samples, 255 had to be excluded due to acute infections or surgical procedures 3 days before the clinic visit, because they could have severely altered concentrations of inflammatory markers. Overall, 5,813 plasma samples remained. For Athens, fibrinogen levels could not be assessed. IL-6, fibrinogen, and CRP showed a moderate correlation, with the Spearman correlation coefficient ranging from 0.41 to 0.51 for all single measurements and from 0.49 to 0.55 for the mean values per patient, with the data of all cities combined. The single cities showed similar correlation coefficients, Barcelona being the only exception, with a low correlation between fibrinogen and IL-6 (*r* = 0.22 and 0.25, respectively).

### Air pollutants

**Figure 1:**
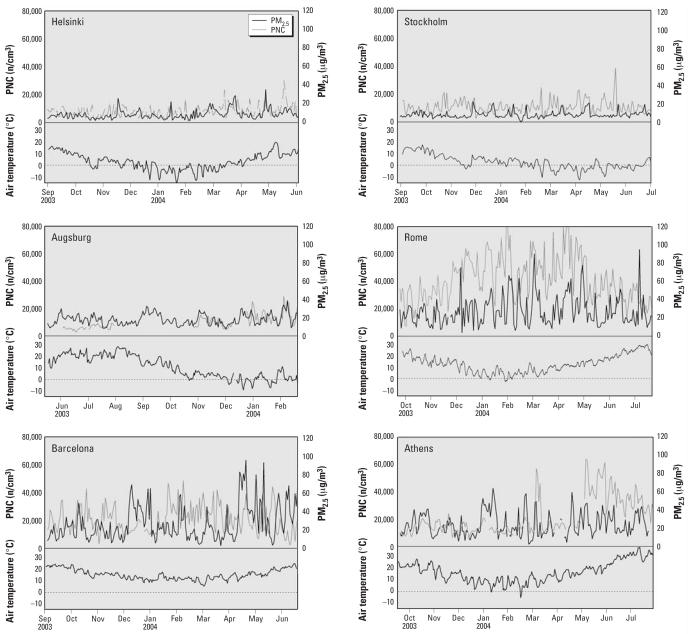
Time series of air pollution (PNC and PM_2.5_) and air temperature in the six European cities of the AIRGENE study.

The 24-hr average concentrations of the pollutants and meteorologic data are given in Table 2. PNC and PM_2.5_ were highest in the southern cities and lowest in Stockholm and Helsinki, whereas Augsburg showed intermediate levels (Figure 1).

### Regression results

**Figure 2:**
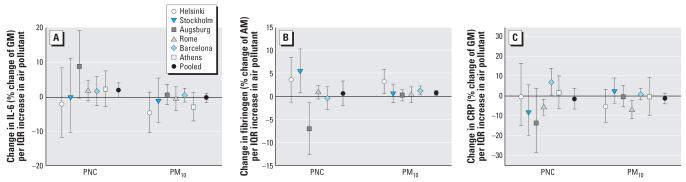
Association between PNC and PM_10_ and blood markers for the single cities for the *a priori* specified lags. (*A*) IL-6. (*B*) Fibrinogen. (*C*) CRP. Abbreviations: AM, arithmetic mean; GM, geometric mean.

**Figure 3:**
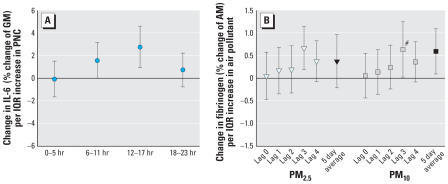
Pooled effects of PNC on IL-6 (*A*) and of PM_2.5_ and PM_10_ (*B*) on fibrinogen, different lags. Abbreviations: AM, arithmetic mean; GM, geometric mean. Error bars indicate 95% CIs. ^#^Heterogeneity between the cities present.

The pooled results for the regression of the three blood markers are summarized in Table 3. IL-6 showed borderline significant increases in association with PNC and NO_2_ with lag 0, one of the two *a priori* specified lags (Figure 2). Because IL-6 showed positive associations for lag 0, we analyzed the 24 hr of air pollution exposure before the blood withdrawal in more detail. PNC results indicate a time response with a slight increase 6–11 hr after an exposure, a clear increase with 12–17 hr after an exposure, and a drop back to the level of 0–5 hr thereafter (Figure 3). Results for 6–11 as well as 12–17 hr for all single cities show clear positive associations, except for Helsinki and Athens (6–11 hr) and Helsinki and Stockholm (12–17 hr).

Fibrinogen was associated with an increase for the 5-day-average exposure of PM_10_. Other pollutants also indicate an increase for the 5-day-averages, but CIs were wide. In addition to the effect for the cumulative exposure, we found an increase for fibrinogen with lag 3 for PM_2.5_ and PM_10_ (Figure 3). For lag 3, results of the single cities show clear positive associations with PM_2.5_ for all cities except for Augsburg, where no association was seen. For PM_10_ and lag 3, results are heterogeneous. Except for Augsburg, all cities present positive associations, with Helsinki being the highest. Associations for PM_2.5_ and PM_10_ for the 5-day-average exposures were positive in all cities.

**Figure 4:**
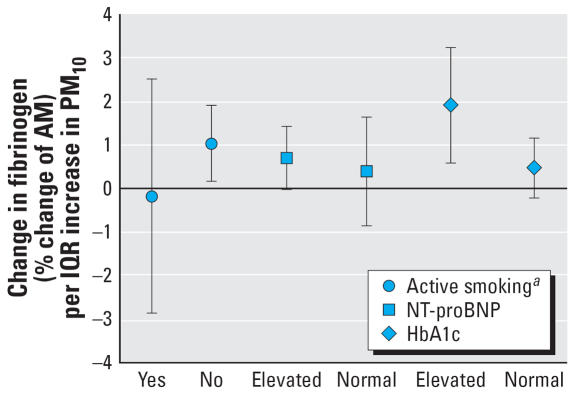
Effects of PM_10_ on fibrinogen (5-day average) as modified by active smoking, levels of NT-proBNP and HbA1c as indicators of left ventricular dysfunction and diabetes, respectively. AM, arithmetic mean. Error bars indicate 95% CIs. ^***a***^Active smokers were only present in Rome and Barcelona; interaction thus calculated only for these two cities.

Analyses of effect modification showed that for fibrinogen associations remained for the 5-day average exposure to PM_10_ for nonsmokers and patients with elevated NT-proBNP and HbA1c levels (Figure 4). Results for the single cities revealed clear positive associations for patients with elevated HbA1c levels in Helsinki and Barcelona, and small positive associations in Augsburg and Rome, whereas no association was found for Stockholm. Helsinki and Barcelona showed clear increases in fibrinogen levels with increased PM_10_ for patients with high NT-proBNP levels, whereas for the other cities only small increases were found. Active smokers were present only in Rome and Barcelona, and interactions with smoking thus were calculated only for these two cities. The combined results are driven mainly by the results from Barcelona, which indicate a strong positive association for nonsmokers. For CRP, no associations between ambient air pollution and serum concentrations were observed for either the *a priori* hypothesized time span or other lags.

### Sensitivity analyses

We performed sensitivity analyses for all blood markers, using selected air pollutants and the *a priori* specified lags (Table 4). For IL-6 and PNC, additional confounders in the model led to a clear positive result, whereas all other models, including the chosen model, were more conservative. For fibrinogen, overall results remained clearly positive and stable with PM_10_. With PNC a strong yet not significant association was found for the model without time-independent covariates. For CRP, results did not change in dependence on the model.

## Discussion

We measured IL-6, fibrinogen, and CRP, three blood markers that indicate an inflammatory response, in MI survivors in six European cities. Pooled results show an increase in IL-6 when concentrations of PNC were elevated 12–17 hr before the clinical visit. Cumulative exposure to PM_10_ was associated with an increase in fibrinogen. No consistent associations could be detected for CRP.

Air pollution seems to affect susceptible subgroups (Goldberg et al. 2001, 2006; Katsouyanni et al. 2001; Pope et al. 2002). We therefore examined MI survivors, a subgroup with an increased risk for readmission to the hospital (von Klot et al. 2005), in six European cities, covering a wide range of gaseous and particulate air pollutants. There is a strong link between inflammation and CHD because factors involved in inflammation and infection seem to play a proatherogenic role, and inflammation has been identified as a potent risk factor for acute ischemic syndromes (Ross 1999). Other risk factors such as cigarette smoking (Danesh et al. 1999: Fröhlich et al. 2003), diabetes (Thorand et al. 2007), or high BMI (Danesh et al. 1999: Thorand et al. 2006) have also been found to be associated with low-grade systemic inflammation, providing a further link between inflammation and acute coronary events.

Previous studies have shown an association between air pollution and blood markers of inflammation and coagulation. We examined IL-6 because of its role in the inflammatory cascade (Woods et al. 2000). IL-6 is produced by different cells in the body, including lymphocytes, monocytes, and endothelial cells. It is thought to play a major role in mediating stimuli from activated macrophages—for example, by smoking. IL-6 is the key cytokine that stimulates the synthesis of all major acute phase proteins, including CRP and fibrinogen (Woods et al. 2000). The latter factor induces an increase in blood viscosity and promotes thrombus formation (Koenig 2003).

In a study in the United Kingdom (Seaton et al. 1999), no significant associations were seen for a 3-day cumulative exposure to ambient PM_10_ and IL-6 levels. At high pollution levels, however, such as in road tunnels or during forest fires, positive associations have been observed (Hilt et al. 2002; van Eeden et al. 2001). Our results indicate an increase in IL-6 within 12 hr after exposure, a requisite first step in the stimulation of the *de novo* synthesis of acute-phase proteins in the liver, triggered by ambient particles.

Fibrinogen, an acute-phase protein, also plays a crucial role in the coagulation cascade. Studies regarding its association with air pollution are inconclusive. It has been shown to increase in association with high levels of ambient particles such as in an air pollution episode (Peters et al. 1997) or in controlled human exposure studies (Ghio et al. 2000). Also, positive associations, such as for PM_10_, have been reported at levels comparable to those measured in the present study (Pekkanen et al. 2000; Schwartz 2001). However, also null associations (Pope et al. 2004; Ruckerl et al. 2006) and even decreases in fibrinogen concentration in association with air pollutants have been reported (Khandoga et al. 2004; Seaton et al. 1999). Our study indicates an increase in fibrinogen for lag 3 and the 5-day cumulative exposure for PM_10_.

CRP, a well-known biomarker of systemic inflammation, has been one of the first acute-phase reactants to be examined in association with air pollution in several studies. Increased concentrations have been shown during an air pollution episode in Germany in healthy men, 45–64 years of age (Peters et al. 2001b) as well as for ambient PM_10_ levels currently present in Europe (Seaton et al. 1999). Additionally, in a panel of CHD patients, an increase in CRP above the 90th percentile was found in association with ambient particles (Ruckerl et al. 2006). Similar analyses did not reveal any effects in our data, which might be attributed to differences in the two panels. The AIRGENE panel consisted of both males and females and was on average slightly younger, but had more severe diseases than the subjects studied previously. On the other hand, the average CRP levels were lower in the AIRGENE panel.

Overall, these studies suggest associations between inflammation and ambient air pollution concentrations, especially particles, although the effects between studies differ for individual data. To date, the reason for the heterogeneity is largely unknown. Different pollution mixtures, underlying medical conditions, treatments or diets with high anti-oxidant levels might be possible explanations.

We observed immediate associations between PNC and IL-6 and cumulative effects between PM_10_ and fibrinogen. This is a surprising finding, which might be attributed to chance, because PM_10_ and PNC were not highly correlated in most cities. It is, however, also possible that their mode of action is different. Ultrafine particles or attached substances might translocate quickly into the bloodstream (Geiser 2002) and lead to the observed changes in IL-6 without having a direct impact on the lung. PM_10_, on the other hand, might only exert an indirect systemic impact by provoking an inflammatory response in the lung that eventually causes oxidative stress, leading to the observed delayed increase in fibrinogen. However, these explanations are highly speculative. Further, PNC and PM_10_ differ not only by size but also by composition and redox activity (Cho et al. 2005), but the implications for the mechanisms are difficult to judge. When the city-specific results are examined, the immediate association between IL-6 and PNC was strongest in Augsburg, whereas the association between PM_10_ and fibrinogen was strongest in Helsinki for the 5-day-average exposure. Because these city-specific estimates were not heterogeneous, this may reflect the expected variation between independent studies. It also might point to differences in measurement error with respect to population average exposures characterized by central monitoring sites.

One possible explanation for the lack of associations between air pollutants and CRP in our data could be the high prevalence of lipid-lowering drugs intake, particularly statins, which have been shown to reduce CRP through inhibition of its hepatic synthesis (Arnaud et al. 2005). Studies have shown that long-term therapy with a statin significantly lowers plasma levels of CRP (Ridker et al. 2001; Sandhu et al. 2005). IL-6, which is produced upstream to the production of CRP in the liver, is not affected by this compound. Also, fibrinogen has been implicated to be reduced by fibrates but not statins (Rosenson et al. 2001). Because the majority of our patients reported an intake of statins, subgroup analyses did not seem reasonable.

Increased concentrations of CRP are known to predict cardiovascular events in healthy subjects (Danesh et al. 2000). Also, elevated levels of IL-6 have been found to be associated with total mortality (Harris et al. 1999) and with risk of future fatal and nonfatal MI (Ridker et al. 2000). Whether the short-term increases in IL-6 and fibrinogen observed in this study actually lead to an increased risk for an acute coronary syndrome, however, remains to be shown. A long-term follow-up study examining cardiovascular end points might help to answer the question whether subjects with elevated levels of inflammatory proteins in response to environmental stimuli have an increased risk of acute ischemic syndromes.

### Strengths and limitations

The study is based on a common protocol and standard operating procedures applied in six European cities. Site visits were conducted to ensure uniform procedures. The analyses of the inflammatory markers were done in one central laboratory, and blinded duplicate samples were measured for quality assurance.

Some of the measured biomarkers (e.g., CRP) are affected by health-related events such as acute infection or surgery (Thomas 2000). We therefore carefully excluded blood samples that might have been strongly influenced by other sources than air pollution before the statistical analyses. Moreover, thorough confounder adjustment was done to rule out the possibility that the detected associations resulted from meteorologic influences or seasonal differences, and repeated measures decreased the chance of confounding by time-independent variables, because each person served as his or her own control.

Even though we included city-specific patient characteristics to account for differences in the panels, the city-specific estimates of the air pollution effects still varied. However, for those results indicating an association between air pollution and inflammation, these variations did not exceed the expected random variation. But it is also quite possible that the air pollution mixture, socioeconomic factors, or genetic background are responsible for these modifications. Indeed, we did observe effect modification for patients with elevated HbA1c and NT-proBNP.

We observed only small changes in the acute-phase response that are not on the scale of a bacterial infection (Thomas 2000) or surgery and presumably do not have any direct clinical relevance. Other factors, such as 10 pack-years of smoking, in comparison, led to increases of 2.7% (95% CI, 1.2–4.3) for IL-6, 1.2% (95% CI, 0.7–1.7) for fibrinogen, and 5.5% (95% CI, 3.0–8.0) for CRP in our data. A higher BMI of 5 kg/m^2^ was associated with significantly higher levels of IL-6 (16%), fibrinogen (3.8%), and CRP (38%). Smoking and overweight may be of concern in subpopulations, whereas air pollution usually affects whole populations and there is generally no voluntary component to the risk. Based on a publication by Cesari et al. (2003), we estimated that the increase in IL-6 we found in association with PNC might lead to a 0.7% (95% CI, –0.06 to 1.5) increased risk of CHD in elderly people without baseline cardiovascular risk. Despite the high prevalence of statin intake, our data still indicate an inflammatory response in association with air pollution. We therefore hypothesize that ambient air pollution might increase plaque vulnerability by these subclinical inflammatory responses.

## Conclusion

Our results indicate an immediate response of IL-6 to ambient air pollution, which might lead to the synthesis of acute-phase proteins, as indicated by increased fibrinogen levels. The lack of detectable associations for CRP may be attributed to a widespread intake of statins in our population, which might suggest a protective effect against environmental, proinflammatory stimuli—an intriguing phenomenon that deserves further study.

## Figures and Tables

**Table 1:** Baseline characteristics of 1,003 MI survivors from six European cities.

Characteristic	Helsinki (*n* = 195)	Stockholm (*n* = 197)	Augsburg (*n* = 200)	Rome (*n* = 134)	Barcelona (*n* = 169)	Athens (*n* = 108)	*p*-Value
Percent male	68.7	70.6	82.0	86.6	83.4	87.0	< 0.0001*
Age [mean years (range)]	64.6 (45–78)	64.0 (38–76)	61.9 (39–76)	62.7 (39–79)	62.1 (37–81)	54.7 (38–75)	< 0.0001**
BMI [mean (range)]	28.6 (19.1–48.9)	27.6 (17.5–43.2)	28.7 (19.1–48.4)	27.7 (19.0–39.4)	28.8 (19.3–43.5)	28.8 (20.8–46.3)	0.0039**
First MI (%)	81.5	85.8	87.5	87.3	86.4	80.6	0.37*
Self-reported history (%)^a^							
Angina pectoris	39.0	47.7	21.0	27.6	29.6	41.7	< 0.0001*
Arrhythmia	31.3	20.8	24.0	23.1	13.0	21.3	0.0029*
Congestive heart failure	14.9	16.2	13.0	6.0	1.8	5.6	< 0.0001*
Hypertension	51.3	49.7	51.0	55.2	46.2	54.6	0.73*
Diabetes	21.0	18.3	17.5	17.2	23.7	21.3	0.63*
Chronic renal disease	3.6	2.0	5.0	5.2	9.5	1.9	0.019#
Asthma	5.1	5.6	4.5	6.7	4.7	0.0	0.0946#
Any respiratory disease	7.2	6.6	10.5	22.4	13.6	6.5	< 0.0001*
Indication of COPD^b^	29.2	30.6	20.5	15.7	27.8	13.9	0.007*
Total cholesterol (mg/dL)^c^ (range)	182.2 (91.1–291.9)	173.4 (96.7–324.7)	181.0 (107.0–316.0)	190.6 (120.0–321.0)	193.2 (119.0–390.0)	195.4 (92.0–293.0)	< 0.0001**
HDL cholesterol (mg/dL)^c^ (range)	54.0 (22.0–119.3)	53.7 (30.9–116.0)	47.9 (24.0–98.0)	43.7 (25.0–87.0)	52.7 (28.0–105.0)	46.1 (24.0–87.0)	< 0.0001**
HbA1c [% (range)]^c^	5.9 (4.7–9.2)	5.0 (3.8–9.9)	5.6 (4.7–9.8)	5.4 (2.8–8.7)	5.1 (3.8–9.8)	5.8 (3.7–10.5)	< 0.0001##
Statins (%)	83	88	89	79	85	73	0.0001*
Lipid-lowering medication (%)	85	89	90	83	86	74	0.0039*
Antithrombotic medication (%)	97	97	99	95	98	93	0.058†
No. of blood samples	1,155	1,168	1,144	741	1,119	486^d^	
IL-6 [mean (pg/mL)]	3.16	2.67	2.60	3.18	3.58	3.19	
GM (range)^e^	2.46 (0.92–19.7)	2.02 (0.48–24.4)	2.16 (0.61–11.8)	2.32 (0.95–61.4)	2.85 (0.76–28.51)	2.52 (0.84–22.40)	
Fibrinogen [mean (g/L)]	3.76	3.53	3.34	3.24	3.99	—	
GM (range)^e^	3.69 (2.68–5.63)	3.44 (2.24–6.11)	3.27 (2.00–6.87)	3.14 (1.94–5.18)	3.91 (2.62–6.02)	—	
CRP [mean (mg/L)]^f^	1.98	2.86	2.26	2.56	3.52	2.52	
GM (range)^f^	1.18 (0.16–12.15)	1.42 (0.16–37.44)	1.18 (0.16–24.65)	1.40 (0.16–15.33)	2.03 (0.33–30.16)	1.32 (0.23–24.25)	

COPD, chronic obstructive pulmonary disease.

a Ever physician diagnosed.

b Evaluated using a questionnaire on symptoms.

c Blood biomarkers determined at local laboratories.

d For fibrinogen N = 0.

e Geometric mean of patients’ geometric mean of repeated measurements.

f Values of CRP < 0.16 could not be measured and were set to 0.16. *p*-Values determined with

* chi-square test,

** ANOVA,

# Fisher’s exact test,

## Median-test,

† Kruskal-Wallis test.

**Table 2:** Twenty-four-hour average concentrations of the ambient air pollution concentrations and meteorologic parameters from six European cities during the AIRGENE study period.^a^

	Helsinki	Stockholm	Augsburg	Rome	Barcelona	Athens
	5 Sep 03–2 Jun 04	30 Aug 03–24 Jun 04	14 May 03–24 Feb 04	20 Sep 03–15 Jul 04	30 Aug 03–16 Jun 04	8 Sep 03–30 Jul 04
Pollutant	Mean (95th)	Mean (95th)	Mean (95th)	Mean (95th)	Mean (95th)	Mean (95th)
PNC (1/cm^3^)	8,534 (15,077)	9,748 (17,578)	11,876^b^ (25,135)	35,450^b^ (69,226)	18,133^b^ (36,526)	20,589^b^ (47,573)
PM_2.5_ (μg/m^3^)	8.2 (19.4)	8.8 (19.1)	17.4 (29.3)	24.5^b^ (54.1)	24.2^b^ (62.7)	23.0^b^ (46.0)
PM_10_ (μg/m^3^)	17.1 (36.1)	17.8 (40.3)	33.1 (56.6)	42.1 (76.0)	40.7^b^ (88.7)	38.5 (64.6)
CO (mg/m^3^)	0.31 (0.46)	0.29 (0.43)	0.58 (1.00)	1.40 (2.47)	0.59 (0.92)	1.48 (3.23)
NO_2_ (μg/m^3^)	28.6 (49.8)	18.6 (32.6)	40.0 (61.2)	67.0 (90.8)	50.5 (79.6)	50.1 (73.0)
NO (μg/m^3^)	12.5 (40.7)	4.9 (15.5)	30.0 (80.4)	65.7 (164.0)	37.7 (88.4)	41.8 (144.6)
SO_2_ (μg/m^3^)	4.2 (10.1)	1.9 (4.9)	3.0 (5.7)	4.1 (9.2)	4.7 (9.6)	10.3 (23.2)
O_3_ [8-hr average ([μg/m^3^)]	46.8 (89.0)	60.6 (96.9)	54.4 (115.3)	45.3 (99.6)	28.2 (76.5)	59.8 (100.2)
Air temperature (°C)	3.1 (14.7)	4.7 (15.1)	10.2 (25.1)	13.4 (23.9)	15.2 (23.2)	17.6 (29.3)
Relative humidity (%)	76 (91)	82 (94)	69 (92)	80 (95)	67 (86)	67 (84)

95th, 95th percentile.

a The study period started 5 days before the first measurement because *a priori* air pollution concentrations up to 5 days before the blood withdrawals were considered.

b Data available on < 95% of the days.

**Table 3:** Effects of air pollution on blood biomarkers per increase in IQR of air pollutant (pooled effect estimates).

		IL-6 (all cities)			Fibrinogen (all cities except Athens)			CRP (all cities)		
Pollutant, IQR	Time before blood withdrawal	% change (GM)	95% CI	*p*-Value heterogeneity	% change (AM)	95% CI	*p*-Value heterogeneity	% change (GM)	95% CI	*p*-Value heterogeneity
PNC^a^										
11852	Lag 0	1.88**	–0.16 to 3.97	0.72	0.40	–0.40 to 1.19	0.54	1.33	–3.05 to 5.90	0.047
11852	Lag 1	–0.67	–2.56 to 1.25	0.64	0.11	–0.69 to 0.91	0.12	–1.52	–4.39 to 1.45	0.19
11852	Lag 2	–2.12**	–4.03 to –0.17	0.055	0.09	–0.71 to 0.90	0.045	–1.63	–6.70 to 3.71	0.019
11003	5-day average	–0.93	–3.37 to 1.56	0.084	0.50	–2.20 to 3.20	0.009	–0.08	–3.78 to 3.75	0.12
PM_2.5_^b^										
11.0	Lag 0	0.46	–0.89 to 1.83	0.26	0.05	–0.48 to 0.58	0.36	0.11	–1.95 to 2.21	0.71
11.0	Lag 1	–0.39	–1.69 to 0.93	0.70	0.17	–0.35 to 0.69	0.55	–0.06	–1.98 to 1.90	0.70
11.0	Lag 2	–0.23	–1.53 to 1.07	0.57	0.20	–0.32 to 0.71	0.26	0.11	–1.80 to 2.06	0.86
8.6	5-day average	0.05	–1.37 to 1.50	0.66	0.38	–0.21 to 0.96	0.21	–0.13	–2.15 to 1.92	0.94
PM_10_^c^										
17.4	Lag 0	–0.34	–1.66 to 0.99	0.45	0.06	–0.43 to 0.55	0.53	–0.71	–2.75 to 1.37	0.16
17.4	Lag 1	–0.69	–1.95 to 0.58	0.43	0.14	–0.35 to 0.63	0.83	–0.63	–2.61 to 1.39	0.23
17.4	Lag 2	–1.59	–3.99 to 0.88	0.0030	0.24	–0.24 to 0.72	0.25	–1.42	–4.23 to 1.47	0.086
13.5	5-day average	–0.87	–2.28 to 0.55	0.15	0.60*	0.10 to 1.09	0.26	–1.35	–3.45 to 0.79	0.19
CO										
0.34	Lag 0	0.57	–0.63 to 1.79	0.95	0.24	–0.45 to 0.92	0.11	–0.01	–1.72 to 1.73	0.18
0.34	Lag 1	0.44	–0.79 to 1.68	0.72	0.32	–0.35 to 1.00	0.38	–1.51	–3.30 to 0.32	0.19
0.34	Lag 2	–2.36	–4.82 to 0.17	0.0054	–0.44	–1.11 to 0.23	0.078	–2.35	–6.84 to 2.36	0.0025
0.31	5-day average	–0.28	–2.53 to 2.02	0.067	0.12	–0.81 to 1.05	0.062	–0.85	–5.37 to 3.90	0.051
NO_2_										
15.9	Lag 0	1.31**	–0.24 to 2.89	0.97	0.05	–0.50 to 0.60	0.84	0.41	–1.93 to 2.81	0.68
15.9	Lag 1	0.93	–0.55 to 2.43	0.78	0.04	–0.49 to 0.57	0.64	1.15	–1.18 to 3.54	0.86
15.9	Lag 2	–1.38	–4.35 to 1.68	0.00024	0.05	–0.71 to 0.80	0.056	–0.28	–4.05 to 3.63	0.0081
10.1	5-day average	–0.19	–3.08 to 2.78	0.0014	0.24	–0.45 to 0.93	0.057	1.40	–0.92 to 3.79	0.19

Abbreviations: AM, arithmetic mean; GM, geometric mean. *A priori* specified lags: IL-6: lag 0 and lag 1; fibrinogen: 5-day average; CRP: lag 2.

a IQR (24-hr, 5-day average), 11852.39408, 11002.9686 n/cm^3^.

b IQR (24-hr, 5-day average), 10.99720847, 8.59343322 μg/m^3^.

c IQR (24-hr, 5-day average), 17.36794382, 13.5380001 μg/m^3^.

* *p* < 0.05;

** *p* < 0.1.

**Table 4:** Sensitivity analyses: results for different models on selected outcomes.

	IL-6 (lag 0)			Fibrinogen (mean of 5-day average)			CRP (lag 2)		
Pollutant, model	Estimate (% change GM)	95% CI	Heterogeneity (*p*-value)	Estimate (% change AM)	95% CI	Heterogeneity (*p*-value)	Estimate (% change GM)	95% CI	Heterogeneity (*p*-value)
PNC									
Main model	1.88	–0.16 to 3.97	0.72	0.5	–2.20 to 3.20	0.009	–1.63	–6.70 to 3.71	0.02
Additional covariates^a^	2.16*	0.08 to 4.29	0.85	0.26	–2.96 to 3.48	0.004	–1.64	–7.02 to 4.05	0.01
No time-independent covariates	1.16	–0.85 to 3.21	0.46	2.77	–0.15 to 5.69	0.001	–1.92	–6.93 to 3.36	0.02
Including risk of potential inflammation 4–7 days before blood withdrawal	1.91	–0.14 to 3.99	0.70	0.48	–2.37 to 3.32	0.01	–1.74	–6.78 to 3.57	0.02
Including seasonal interaction (season = winter)	1.97	–0.11 to 4.09	0.61	0.35	–0.80 to 1.51	0.02	–1.18	–6.59 to 4.55	0.01
PM_10_									
Main model	–0.34	–1.66 to 0.99	0.45	0.60*	0.10 to 1.09	0.26	–1.42	–4.23 to 1.47	0.086
Additional covariates^a^	–0.16	–1.48 to 1.17	0.53	0.81*	0.17 to 1.45	0.19	–1.08	–3.05 to 0.92	0.11
No time-independent covariates	–0.64	–1.94 to 0.69	0.44	0.36	–0.77 to 1.48	0.02	–2.04	–5.05 to 1.07	0.04
Including risk of potential inflammation 4–7 days before blood withdrawal	–0.35	–1.66 to 0.99	0.44	0.75	0.12 to 1.38	0.34	–1.43	–4.21 to 1.43	0.09
Including seasonal interaction (season = winter)	–0.15	–1.71 to 1.42	0.40	0.78	–0.02 to 1.57	0.50	–1.33	–3.61 to 1.00	0.55

Estimates for CRP and IL-6 are expressed as percent change in expected geometric mean (GM); estimates for fibrinogen are expressed as absolute change in expected mean, expressed as percent of overall arithmetic mean (AM).

a Additional covariates included time-independent variables present in at least two cities, hour of blood withdrawal, and relative humidity.

* *p* < 0.05.

## Appendix 1: The AIRGENE study group comprises the following partners

1. ***GSF-National Research Centre for Environment and Health, Institute of Epidemiology (Neuherberg, Germany):*** A. Peters, M.-A. Bind, I. Brueske-Hohlfeld, H. Chavez, J. Cyrys, U. Geruschkat, H. Grallert, S. Greven, A. Ibald-Mulli, T. Illig, H. Kirchmair, S. von Klot, M. Kolz, M. Marowsky-Koeppl, M. Mueller, A. Ossig, R. Rückerl, A. Schaffrath Rosario, A. Schneider, G. Walter, H.-E. Wichmann; Institute of Health Economics and Health Care Management (Neuherberg, Germany): R. Holle, H. Nagl; KORA Study Center (Augsburg, Germany): I. Fabricius, C. Greschik, F. Günther, M. Haensel, U. Hahn, U. Kuch, C. Meisinger, M. Pietsch, E. Rempfer, G. Schaich, A. Schneider, I. Schwarzwälder, B. Zeitler; KORA Myocardial Infarction Registry (Augsburg, Germany): H. Loewel; Ludwig-Maximilians University (Munich, Germany): H. Küchenhoff.
2. ***University of Ulm, Department of Cardiology (Germany):*** W. Koenig, N. Khuseyinova, G. Trischler.
3. ***Local Health Authority RM E, Department of Epidemiology ASL RME (Rome, Italy):*** F. Forastiere, P. Compagnucci, F. Di Carlo, M. Ferri, A. Montanari, C. Perucci, S. Picciotto, E. Romeo, M. Stafoggia; Catholic University (Rome, Italy): R. Pistelli, L. Altamura, M.R. Andreani, F. Baldari, F. Infusino, P. Santarelli; Hospital San Filippo Neri: A.P. Jesi; Italian National Institute of Health (Rome, Italy): G. Cattani, A. Marconi.
4. ***National Public Health Institute (KTL) (Kuopio/Helsinki, Finland):*** J. Pekkanen, M. Alanne, H. Alastalo, T. Eerola, J. Eriksson, T. Kauppila, T. Lanki, P. Nyholm, M. Perola, V. Salomaa, P. Tiittanen; Helsinki University Central Hospital: K. Luotola.
5. ***Institute of Environmental Medicine, Karolinska Institute (Stockholm, Sweden):*** Department of Occupational and Environmental Health, Stockholm County Council (Stockholm, Sweden); Department of Cardiology, Stockholm South Hospital (Stockholm, Sweden): T. Bellander, N. Berglind, K. Bohm, R. Härdén, E. Lampa, P. Ljungman, F. Nyberg, B. Ohlander, G. Pershagen, M. Rosenqvist, T. Larsdotter Svensson, E. Thunberg, G. Wedeen.
6. ***Municipal Institute of Medical Research (Barcelona, Spain):*** J. Sunyer, M. Covas, M. Fitó, M. Grau, B. Jacquemin, J. Marrugat, L. Muñoz, G. Perelló, E. Plana, C. Rebato, H. Schroeder, C. Soler.
7. ***University of Athens Medical School, Department of Hygiene and Epidemiology (Athens, Greece):*** K. Katsouyanni, A. Chalamandaris, K. Dimakopoulou, D. Panagiotakos; Department of Cardiology (Athens, Greece): C. Stefanadis, C. Pitsavos, C. Antoniades, C. Chrysohoou, J. Mitropoulos.
8. ***University of Helsinki, Department of Physical Sciences (Helsinki, Finland):*** M. Kulmala, P. Aalto, P. Paatero.
